# A Concise History of the Principal Discoveries in Anatomy

**Published:** 1800-01

**Authors:** Curt Sprengel


### *A Concise History of the Principal Discoveries in Anatomy.*

[Extracted from the original German of Prof. CURT. SPRENGEL's History of Medicine, Vol. III. Sect. XI.—Continued from our preceding Volume, p. 463.]

AFTER having pointed out the individual merits of the most celebrated anatomists in that century, given a general account of their respective works, and examined the progressive discoveries and improvements made in *osteology*, *myology*, and *angiology*, particularly relative to the circulation of the blood, and the knowledge of the lymphatics, we now proceed to the no less important subject of *splanchnology*.

§ 24. The nature of the peritoneum and its processes puzzled the anatomists of this century considerably, and yet they could not attain to any degree of accuracy. MASSA described it, and likewise the mode of exenterating, but both unsatisfactorily.* It

* Introduct. f. 12. b.

It was generally believed, that the peritoneum were perforated in the annulus abdominis, and that, at least, none of its processes accompanied the descent of the testes. Even VESALIUS was of this opinion.* SYLVIUS, his vehement adversary, was at least this time perfectly right, in opposing the sanctioned error, and proving that the peritoneum frequently is not perforated in those places. It is singular, however, that this strenuous vindicator of the old school did not venture to draw any general results from his own observations, but would rather ascribe to praeternatural causes what must always be considered as the common course of nature, † FALLOPIUS likewise explains the origin of ruptures, especially in the female sex, from the elongation of these processes of the peritoneum. ‡—The duplicatures of the peritoneum around different viscera are well described by COLUMBUS. § VESALIUS was the first who accurately represented the duplicatures, which constitute the omentum, and their continuation with the stomach, the spleen, and the colon, demonstrating particularly that the omentum in men never descends so much, as GALEN observed in quadrupeds: he gave a good description of the *appendices epiploicæ* of the colon. ‖ But FABRICIUS wrote a very full account of the omentum,** with a detail of its origin from the region of the spine; its oblique descent towards the stomach; its connection with the *lobulus Spigelii* of the liver; its adhesion to the colon and to the spleen, and the retortion of one lobe upwards to the umbilical region.

With respect to the *stomach*, VESALIUS corrected GALEN's error, who maintained, that the neighbourhood of the lower orifice of the stomach was closed by a glandular substance. VESALIUS admitted its existence in dogs, but at the same time he first described the real nature of the orifice, and particularly the sphincter pylori††, which afterwards was copied by GUIDI ‡‡. As to the *liver*, the processes, which the peritoneum sends off to the liver, were already observed by BERENGAR, though he considered them as a seperate membrane, and did not sufficiently distinguish them. §§ The old idea of the liver being divided into four or five large lobes, which undoubtedly originated from dissections of dogs, was still retained by BERENGAR. ‖‖ But MASSA adopted one single fissure, which, however, did not divide the whole

* Lib. v. c. 2. p. 414.

† *Sylv.* observ. f. 71. b

‡ *Fallop* observ. p. 408.

§ Lib. xi. c. 11. p. 433.

‖ *Vesal.* de radic. chyn. p. 643.

** *Fabric.* de omento, p. 123, 124, s.

†† *Vesal.* lib. v. c. 3. p. 417.

‡‡ *Vid.* lib. v. c. 5. p. 238.

§§ Comment. in *Mundin*, f. 144, b.

‖‖ *Ib.* f. 145. a.

whole liver, but only formed two lobes.* VESALIUS treats at large of this subject, and observes at the same time, that the form of the liver, and its divisions, are subject to several deviations, and do not constantly present the same appearance.† SYLVIUS admitted, that there were only two principal lobes of the liver, but that two smaller ones were frequently observed, which consequently made four.‡ PUTEUS pretended that he had found five lobes in the liver of a Prince of Savoy: § but COLUMBUS sufficiently refuted this opinion. ||—On the biliary ducts, ZERBI made the observation, that they partly convey the bile into the stomach. ¶ VESALIUS had likewise observed the same, but justly considered it as a deviation. ** FALLOPIUS appears, notwithstanding, to doubt the truth of this observation, as he had never made it himself. †† The valves, which DU LAURENS imagined he had found in the ductus communis choledochus,‡‡ have not been verified by experience, as little have the immediate ducts from the liver to the gall-bladder, which JASOLINI, a pupil of INGRASSIAS, described and even represented in engravings. §§ Probably these figures were taken from the livers of fishes and birds, in which we find such ducts. ||||

§. 25. It has now and then been supposed, that the anatomists of the sixteenth century, knew the *pancreas*, as they make use of the name; but what they denominate thus, is nothing but a congeries of glands, in the centre of the mesentery, which WINTHER VON ANDERNACH describes, in a manner not at all correspondent to our pancreas.¶¶ SYLVIUS gives a similar description of this viscus.*** With FALLOPIUS also, this name signifies a collection of small glands in the middle of the mesentery, for the purpose of transmitting the splenic vein from the spleen to the vena portarum.††† VESALIUS‡‡‡ and COLUMBUS§§§ describe this viscus in a similar manner; the former even asserts, that the pancreas is covered with the duplicature of the omentum.|||||| FALLOPIUS carefully examined the inner coats of the intestines, and described the folds of which they are formed.¶¶¶ BERENGAR first accurately described the *cæcum*,**** with its adhering process, and made the remark

* Int oduct. f. 27. a.

† *Vesal.* lib. v. c. 7. p. 432.

‡ *Sylv.* isag. p. 70 —Vesan. calumn. depuls. p. 111.

§ Apolog. pro Galen. i. 135. b.

|| Lib. vi. p. 299.

¶ *Zerb.* anatom. p. 34

** Lib. v. c. 8. p. 436.

†† Obs. p. 145.

‡‡ Lib. vi. c. 20. p. 471.

§§ *Jasolini* de poris choledochis, p. 55. (4to. Neap. 1577.)

|||| *Haller*. element. physiol. vol. vi. p. 532.—compare *Fallop*. obs. p. 415.

¶¶ Instit. anat. lib. i. p. 26.

*** Isagog. f. 179. a.

††† Obs. p. 414.

‡‡‡ Lib. v. c. 4. p. 423.

§§§ Lib. xi. c. 6. p. 424.

|||||| L. c. p. 422.

¶¶¶ Obs. p. 412.

**** Comment. in *Mundin*. f. 115. a.

mark confirmed by MORGAGNI;* that sometimes no cavity is observed in this appendage, which he believes to be the case, particularly in persons who have habituated themselves to excessive eating. VESALIUS corrected the erroneous opinion, which prevailed since the time of GALEN, that the *cæcum* constituted so spacious a cavity, as to induce anatomists to consider it as a second stomach. He showed, that the process that adhered to the *cæcum*, was smaller in men than in carnivorous animals, from which, apparently, GALEN borrowed his description.† MASSA‡ and SYLVIUS,§ likewise represented the appendage of the colon, better than their predecessors. The latter, notwithstanding, seduced by his predilection for GALEN, explained the cases which occurred to him, as preternatural. FALLOPIUS compares the cæcum of men, in regard to its small size, to a lumbricus, and reckons it as a part of the colon.‖ FABRICIUS very properly distinguishes the size and situation of the *processus cæci vermiformis* in men, and in different kinds of animals; but makes it also a part of the colon.¶

This idea, that the cæcum appertained to the colon, probably arose from the circumstance, that, by closer examination, the process of the former, proved to be so uncommonly small, in comparison to the description of the ancients; hence, also the reason, why the valve in the commencement of the colon, which was early discovered, was referred to the cæcum. Besides ACHILLINI, who speaks rather obscurely of this famous valve,** LAGUNA describes it a little more distinctly;†† after him FALLOPIUS, from observations made on apes;‡‡ then VAROLI,§§ who arrogates to himself the discovery of it; then POSTHIUS,‖‖ who saw it at Montpellier, where he dissected under RONDELET; after him, SALOMON ALBERTI, in the year 1563;¶¶ and lastly BAUHIN, who observed this valve in 1579. The two last mentioned anatomists first furnished us with representations of that valve.*** Although DU LAURENS informs us, that BAUHIN is the discoverer, it is well ascertained, that he only can claim the merit of having given the first detailed and good description of this valve; and that HALLER

---

* De sedib. et. caus. morb. ep. LXVII. n. 11.

† Lib. v. c. 5. p. 426.

‡ Introduct. p. 21.

§ Obs. f. 71. b.

‖ Instit. anat. p. 433.

¶ De intestin. p. 147.

** Annotat. in *Mundin.* Anatom.

†† *Lacun.* Anatom. method. p. 16.

‡‡ In a MS. never printed, and of which *Blumenbach* gives an account in his Medicin. Bibliothek, (in German) Vol. I. p. 373.

§§ Anatom. lib. ii. c. 3. p. 70.

‖‖ Obs. in *Columb.* p. 504.

¶¶ Hist. part. corp. hum. p. 49, 174.

*** Theatr. anat. lib. i. c. 17. p. 63, 64. Institut. anat. p. 40.

HALLER is very wrong,* in referring ALBERTI's observation to the year 1589,† when the latter expressively says, he has observed this valve, twenty-one years prior to that period, dating the preface of his work with the year 1585. Nor does HALLER's quotations of GUIDI,‡ give a better test of the existence of this valve, since that only refers to the folds, formed by the inner coat of the intestines. PICCOLHUOMINI described the valve, the next after BAUHIN; § as FABRICIUS likewise did ||

§ 26. The uropoetic vessels were first examined by BERENGAR. His object was to decide the question, chiefly suggested by ZEBRI, whether the urine was percolated and secreted in the kidneys, in a manner similar to a filtre. In order to ascertain the fact, BERENGAR instituted the following experiment: he fixed a tube in the emulgent arteries, and injected the bason of the kidneys with warm water, but nothing passed into the ureters; he then dissected the kidney, and found that the finest branches of the emulgent arteries did by no means inosculate with the branches of the tubuli uriniferi, as was supposed before him; but that they diverge in the papillous substance: he also properly described these papillæ. ¶ Next to BERENGAR, the observations of EUSTACHIUS on the kidneys, have much promoted the true knowledge of these viscera. In determining the situation of both the kidneys, he deviated from all his predecessors; and maintained, that the right kidney seldom lay higher than the left; that their situation is for the most part constant, and sometimes even the left kidney a little higher than the right.** VAROLI acceded to this opinion. †† EUSTACHIUS likewise first described the glands, situated above the kidneys, now known by the name of *glandulæ renales*, or *renes succenturiati*; ‡‡ he accurately demonstrated the adipose membrane of the kidneys,§§ and justly censured those authors, who, misled by zootomy, adopted several cavities in the substance of the kidneys; |||| he was more succesful with BERENGAR's experiment of injecting the emulgent arteries; the liquor passed into the ureter; which

* *Laurent.* Hist. anatom. lib. vi. c. 14. p. 429.

† Element. physiol. Vol. vii. p. 132.

‡ *Vid.* lib. v c. 5. p. 237

§ Anatom. prælect. p. 86.

|| De intestin. p. 142.

¶ Comment. in *Mundin.* f. 178. b. 179. a.—It ought to be remembered on this occasion, that BERENGAR could have no idea of the peculiar function of the veins, as he ascribed to them secretion.

** *Eustach.* de renum struct. c. 12. p. 31.

†† Varoli anatom. lib. iii. c. 7. p. 79.

‡‡ *Eustach.* l. c.

§§ *Ib.* c. 4. p. 11.

|||| *Ib.* c. 9. p. 24.

which induced him to decide in favour of the ancients, that the urine was secreted from arterial blood*. He demonstrated admirably, that the substance of the kidneys is interwoven with many nerves, is exquisitely sensible, and that no valve exists at the orifice of the ureters;† thus refuting inveterate prejudices. Fallopius likewise made the important discovery of tubes in the papillous substance of the kidneys,‡ which had erroneously received its name from Bellini. Massa§ at first, and after him, Eustachius|| more perfectly demonstrated, that the ureters consist only of one membrane. For the discovery of the *sphincter vesicæ*, we are indebted to Fallopius:¶ the description of this muscular body by Vesalius being such, that one might rather take it for a lateral muscle of the prostrate gland, than a sphincter.** But Varoli represents the sphincter properly, after Fallopius.††

§ 27. On the parts within the cavity of the thorax, we shall only remark the following. The great process of the pleura, called *mediastinum*, was first accurately examined, and distinctly described by Vesalius. He particularly pointed out the error of the ancients, who admitted a cavity, formed by the septum, and in which a part of the lungs was contained. This cavity, says Vesalius, really exists in different animals, endowed by Nature with more divided lungs; but in men, the interstice between the two membranes of the mediastinum is partly filled up with cellular texture, and this cavity only relates to the region under the sternum,‡‡ where it can be well demonstrated by inflation. Eustachius, in his drawing of the mediastinum, has erroneously represented its membranes as exactly parallel, whereas they join in the anterior and inferior part, but are separated in the superior and posterior part, by the thymus gland.§§ Vesalius demonstrated the single structure of the pleura, and thus corrected the error of Galen, who had enriched it with a double membrane. But Columbus,|||| who, like Galen, took the external cellular texture for one of the membranes of the pleura, rejected the opinion of Vesalius, and the erroneous idea of Columbus has been adopted and spread, till it was completely refuted by Winslow.¶¶ Vesalius so frequently

* *Eustach.* c. 37. p. 95.

‡ *Fallop.* obs. p. 415.

§ Introduct. p. 22.

|| L. c. c. 19. p. 52.

¶ *Fallop.* obs. p. 412.

** Lib. v. c. 11. p. 445.

†† Anatom. lib. iii. c. 7. p. 81.

‡‡ Lib. vi. c. 3. p. 495. seq.

§§ *Eustach.* tab. xv. f. 1. Compare *Haller* elem. physiol. vol. i. p. 261.

|||| Columb. lib. xi. c. 3. p. 414.

¶¶ Exposition anatom. de la structure du corps, tom. iv. p. 86.

frequently obferved the lungs adhere to the pleura after death, that he was thence induced to conjecture *ligaments of the lungs*.*

In the *larynx*, BERENGAR difcovered both the *arytænoid cartilages*, as only one was known before his time; and likewife a mufcular fubftance, probably the *arytænoid gland*.† Nay, VESALIUS and FABRICIUS defcribe even two thyroid glands, either as a deviation in the human fpecies, or from their obfervations on animals.‡ The firft good defcription of the *ventriculus laryngis* was given by COLUMBUS.§ The mufcular fibres which occupy the pofterior part of the larynx, and affift the office of the anterior cartilages, were confidered as ligaments by the anatomifts of thofe times.||

§ 28 On the parts in the cavity of the mouth, we fhall here mention only the remark of FALLOPIUS, that the *uvula* does not belong to the foft palate, as we were taught by the ancients, nor does it ferve to modulate the voice, as was then the general opinion.¶ All the anatomifts of the fixteenth century were acquainted with the orifice of the *Warthonian* falival duct under the tongue, which had been defcribed by GALEN. We find an account of it in the works of ACHILLINI** and BERENGAR.†† BAUHIN feems to have difcovered the *Stenonian* duct.‡‡

In the human eye, the organs of fecretion and the lachrymal ducts were now firft examined with accuracy. BERENGAR knew the *puncta lachrymalia* to be orifices of the *cornua lachrymalia*. He obferved in them a flocculent membrane, which ferved to reftrain the tears; from thefe lachrymal ducts, according to him, the tears flow through the ducts of the nafal bones into the noftrils and fauces, which is the reafon why we perceive the odour, and fometimes the flavour of this fluid.§§ ZERBI knew before this time the puncta lachrymalia;|||| but the firft anatomifts of this century, nay, even COLUMBUS, mifled by zootomy, adopted a double lachrymal gland in the human eye, miftaking the caruncle for the fecond, although it has no connection with the ducts between which it is fituated. VESALIUS,

---

* Lib. vi. c. 7. p. 504. † Comment. in *Mundin*. f. 393. b.

‡ *Vefal.* lib. ii. c 22. p. 214. *Fallop.* obf. p. 452.

§ *Columb.* lib. i. c. 13. p. 83.

|| *Vid.* lib. vi. c. 4. p. 280.—Laurent. lib. iii. c. 9. p. 193.

¶ Obf. p. 382.—Inftit. anat. p. 452. ** Annotat. in *Mundin*. p. 11.

†† Commentar. in *Mundin*. f. 401. b.

‡‡ Theatr. anat. lib. iii. c. 89. p. 520.

§§ *Berengar*. comment. in *Mundin*. f. 467. b.

|||| Anatom. f. 121. b.

VESALIUS* firſt removed this error: He diſtinguiſhed the great lachrymal gland, ſituated above the external angle of the orbit, from the caruncle; determined the uſe of the latter, by maintaining that it ſerved to convey the tears into the lachrymal ducts, and to ſeparate the eye-lids; he alſo deſcribed the ſemilunar membranes which expand before it, and form in ſome animals a third eye-lid.† MASSA likewiſe carefully diſtinguiſhed theſe two bodies ‡ FALLOPIUS ſtill more exactly repreſented the courſe of the cornua lachrymalia into the ſaccus lachrymalis, and thence into the ductus naſalis; § as likewiſe TAGLIACOZZI, who vindicated the true deſtination of the lachrymal caruncle.‖ GUIDI mentions the rudiment of a third eye-lid in man, by the name of a ſmall cartilage; ¶ and SALOMON ALBERTI** adopted the diſcovery of his predeceſſors, and deſcribed admirably well, for the age in which he wrote, the ſecretory organs of the tears. FABRICIUS, after ſuch predeceſſors, ought to have given a more accurate account of the parts before-mentioned; he is therefore leſs intitled to our praiſe.††

The tunica albuginea of the eye, the more ancient anatomiſts ſuppoſed to be derived from the perioſteum of the orbit: MASSA was the firſt who corrected this error.‡‡ FALLOPIUS firſt deſcribed the ciliary proceſſes, and maintained that they ought not to be called membranes: He diſcovered the capſule of the vitreous humour, or hyaloidea *(tunica hyalina)*, and determined more accurately the figure of the cryſtalline lens.§§ VESALIUS was ſtill undetermined both as to the figure, and the true uſe of this ſubſtance.‖‖ He alſo adopted an equal proportion of the diameters of the humours in the eye, a conjecture in which he was refuted by ARANZI.¶¶

[To be continued in our next Number.]

* Lib. x. p. 399. 400.

† Exam. obſerv. *Fallop.* p. 826.

‡ Introduct. p. 91.

§ Obs. p. 426.

‖ De curtor. chirug. lib. i. c. 7. p. 24.

¶ *Vid.* lib. ii. c. 10. p. 69.

** *Alberti* Orationes. 8. Norib. 1585.

†† *Fabric.* de oculo, p. 198.

‡‡ Introduct. p. 92.

§§ Fallop. obſ. p. 427.

‖‖ Lib. vii. c. 14. p. 559.

¶¶ Obs. c. 20. p. 69.

HINTS

